# Viral Sequestration of Antigen Subverts Cross Presentation to CD8^+^ T Cells

**DOI:** 10.1371/journal.ppat.1000457

**Published:** 2009-05-29

**Authors:** Eric F. Tewalt, Jean M. Grant, Erica L. Granger, Douglas C. Palmer, Neal D. Heuss, Dale S. Gregerson, Nicholas P. Restifo, Christopher C. Norbury

**Affiliations:** 1 Department of Microbiology and Immunology, Pennsylvania State University, Milton S. Hershey College of Medicine, Hershey, Pennsylvania, United States of America; 2 Surgery Branch and Center for Cancer Research, National Cancer Institute, National Institutes of Health, Bethesda, Maryland, United States of America; 3 Department of Ophthalmology, University of Minnesota, Minneapolis, Minnesota, United States of America; Oregon Health & Science University, United States of America

## Abstract

Virus-specific CD8^+^ T cells (T_CD8+_) are initially triggered by peptide-MHC Class I complexes on the surface of professional antigen presenting cells (pAPC). Peptide-MHC complexes are produced by two spatially distinct pathways during virus infection. Endogenous antigens synthesized within virus-infected pAPC are presented via the direct-presentation pathway. Many viruses have developed strategies to subvert direct presentation. When direct presentation is blocked, the cross-presentation pathway, in which antigen is transferred from virus-infected cells to uninfected pAPC, is thought to compensate and allow the generation of effector T_CD8+_. Direct presentation of vaccinia virus (VACV) antigens driven by late promoters does not occur, as an abortive infection of pAPC prevents production of these late antigens. This lack of direct presentation results in a greatly diminished or ablated T_CD8+_ response to late antigens. We demonstrate that late poxvirus antigens do not enter the cross-presentation pathway, even when identical antigens driven by early promoters access this pathway efficiently. The mechanism mediating this novel means of viral modulation of antigen presentation involves the sequestration of late antigens within virus factories. Early antigens and cellular antigens are cross-presented from virus-infected cells, as are late antigens that are targeted to compartments outside of the virus factories. This virus-mediated blockade specifically targets the cross-presentation pathway, since late antigen that is not cross-presented efficiently enters the MHC Class II presentation pathway. These data are the first to describe an evasion mechanism employed by pathogens to prevent entry into the cross-presentation pathway. In the absence of direct presentation, this evasion mechanism leads to a complete ablation of the T_CD8+_ response and a potential replicative advantage for the virus. Such mechanisms of viral modulation of antigen presentation must also be taken into account during the rational design of antiviral vaccines.

## Author Summary

Understanding the pathways by which protective immunity is mediated against viral pathogens is essential to allow the design of effective vaccines. No effective vaccine has been designed to activate killer cells of the immune system expressing CD8, although CD8^+^ T cells are the most effective cells at modulating anti-viral immunity. We have studied the process that activates the CD8^+^ T cell to better understand how the cells are triggered so future vaccines might readily activate these cells. CD8^+^ T cells are activated following recognition of small peptides derived from a virus that binds to a cell surface MHC molecule. Many viruses have evolved to prevent the presentation of these peptide-MHC complexes to CD8^+^ T cells. However, the immune system avoids these viral “evasion” mechanisms by allowing virus-derived peptides to be generated from viral proteins that are taken up by uninfected cells, a process termed “cross presentation”. We have shown that a poxvirus can specifically prevent the presentation of its proteins by uninfected cells, the first demonstration of evasion of cross presentation. This knowledge is vital in the use of certain viral vectors during vaccine design and adds to the numerous ways in which viruses can evade the immune system.

## Introduction

CD8^+^ T cells (T_CD8+_) play important roles in host elimination of pathogens, tumors and transplanted tissues. Virus-specific T_CD8+_ recognize major histocompatibility complex (MHC) class I molecules bound to peptides derived from viral proteins [1]. These peptide-MHC complexes can be generated via two spatially distinct pathways. Virus-infected cells present peptides derived primarily from a subset of viral proteins that are rapidly degraded in a process known as direct presentation [2]. Alternatively, long-lived protein substrates may be transferred from virus-infected cells to pAPC where they are processed and presented by uninfected cells via the cross-presentation pathway [3]. The extent to which the direct or cross-presentation pathways contribute to the induction of virus-specific T_CD8+_ *in vivo* remains controversial [4]. Many pathogens have evolved mechanisms to modulate or evade the direct-presentation pathway [5], implying that such mechanisms may confer a survival advantage. Cross presentation is generally thought to compensate when direct presentation is blocked, allowing the generation of specific T_CD8+_ targeting such pathogens [5]. Here we delineate a unique mechanism of viral immune evasion whereby viral antigen is prevented from entering the cross-presentation pathway.

We investigated the pathways used for presentation of vaccinia virus (VACV) antigens driven by late promoters. Recombinant antigens driven by VACV late promoters, which are active only following DNA replication, stimulate poor or undetectable T_CD8+_ responses as compared with the response to identical antigens driven by early VACV promoters [6]. This reduced response occurs despite production of much larger quantities of late promoter-driven antigen both *in vitro* and *in vivo*. The inability of late VACV promoter-driven antigen to stimulate T_CD8+_ responses has been correlated to an abortive *in vitro* infection of pAPC in which late antigens are not produced and so direct presentation cannot occur [7]. Here, we demonstrate that despite the availability of the cross-presentation pathway for initiation of an antiviral T_CD8+_ response the late VACV promoter driven antigen cannot enter the cross-presentation pathway. We provide evidence of a mechanism that is dependent upon sequestration of antigen during the poxvirus life cycle and which is specific for the cross-presentation pathway within pAPC. These data are the first to describe an evasion mechanism of the cross-presentation pathway that in the absence of the direct-presentation pathway leads to a complete ablation of the T_CD8+_ response and a likely replicative advantage for the virus.

## Results

**Figure 1:**
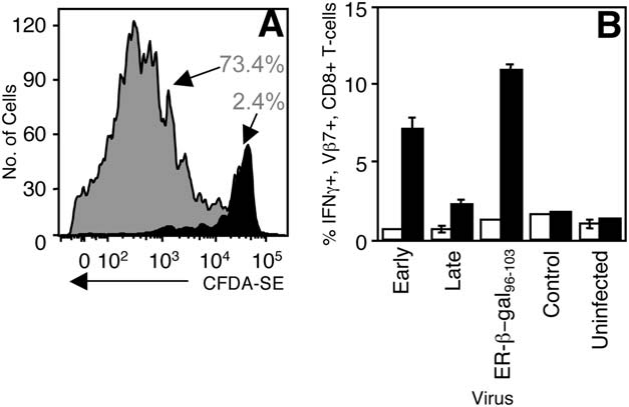
Late VACV promoter-driven antigen does not elicit a T_CD8+_ response. *In vivo* proliferation (A) or *ex vivo* effector function (B) of adoptively transferred β-gal specific BG1 TCR transgenic T_CD8+_ was examined in response to immunization with rVACV-β-gal-Early [(A), gray] or rVACV-β-gal-Late [(A), black] or as shown (B). Proliferation was measured by dilution of CFDA-SE (A) and numbers shown represent the percentage of cells that have diluted the dye 2 days after immunization. *Ex vivo* effector function (B) was measured by quantifying production of IFN-γ in the presence (black) or absence (white) of β-gal_96–103_ peptide.

In order to directly study the effects of driving antigen expression with early or late VACV promoters following infection, we used recombinant viruses in which the early p7.5 or late p11 promoter drive expression of a model antigen. We used β galactosidase (β-gal) as a model antigen as it contains well-defined MHC class I binding determinants and its activity can be readily measured by enzymatic methods even when present in low quantities. We measured proliferation of adoptively transferred BG1 TCR transgenic T_CD8+_ (specific for β-gal_96–103_-K^b^ complexes) [8] in response to immunization with VACV expressing β-gal driven by the p7.5 (rVACV-β-gal-Early) or p11 (rVACV-β-gal-Late) promoters. The BG1 T_CD8+_ did not proliferate (Fig. 1A) or acquire effector activity (Fig. 1B) upon immunization with rVACV-β-gal-Late and did not accumulate above background levels following immunization with a control VACV (data not shown). Proliferation of BG1 T_CD8+_ in mice immunized with rVACV-β-gal-Late could be stimulated following subsequent immunization with adenovirus encoding β-gal (data not shown). Thus, late promoter-driven β-gal does not stimulate T_CD8+_ responses, and the lack of a T_CD8+_ response does not result from tolerance induced by high dose late promoter-driven antigen.

**Figure 2:**
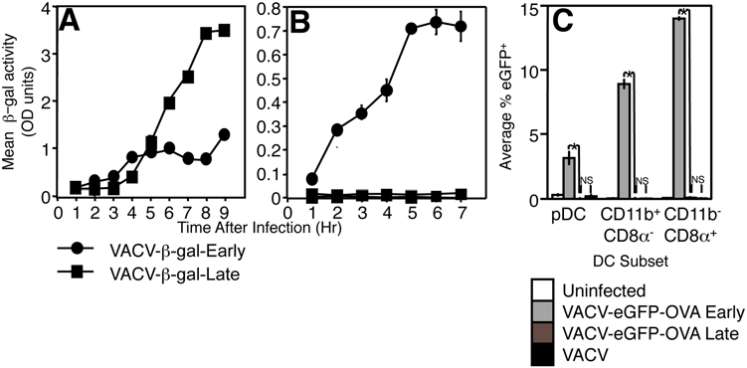
VACV-infected DC do not produce late antigens. Production of β-gal was measured in TAg-β_2_m_neg_ fibroblasts (A) or BMDC (B) infected with rVACV-β-gal-Early (•) or rVACV-β-gal-Late (▪). Note the 5 h time point in (A) at which production of early and late promoter-driven β-gal is at equivalent levels. (C) Production of eGFP was measured in pDC, CD11b^+^ CD8α^−^ DC, or CD11b^−^ CD8α^+^ DC subsets that were uninfected (white bars) or infected with rVACV-eGFP-OVA-Early (light gray bars), rVACV-eGFP-OVA-Late (dark gray bars), or VACV-WR (black bars). *P<0.001, NS = Not Significant (P>0.05).

The reduced immunogenicity of recombinant antigens driven by late VACV promoters has been correlated to a lack of activity of these promoters in pAPC, such as macrophages [9] and dendritic cells [7] *in vitro*. To determine whether late VACV promoters are functional in various cell types we measured β-gal production in a fibroblast cell line or in bone marrow-derived dendritic cells (BMDC) infected with either rVACV-β-gal-Early or rVACV-β-gal-Late using a chromogenic β-gal substrate. Our limit of detection using a chromogenic β-gal substrate is 10^−8^ mg/mL of β-gal (Fig. S1). Figure 2A demonstrates typical expression of β-gal from each virus in fibroblasts. rVACV-β-gal-Early produced a linear accumulation of β-gal almost immediately following infection, while β-gal from rVACV-β-gal-Late is not detectable until >3 h post infection. β-gal produced from rVACV-β-gal-Late rapidly accumulates in much greater quantities than that from rVACV-β-gal-Early, with equivalent levels of β-gal present after 5 h of infection.

In contrast to β-gal production in fibroblasts, expression of β-gal from rVACV-β-gal-Late was undetectable in BMDC (Fig. 2B) while β-gal production from rVACV-β-gal-Early occurred rapidly after infection. As our limit of detection was 10^−8^ mg/mL we can conclude that β-gal production was lower than 10 attograms/cell (10^−18^ g/cell) in BMDC. DC are phenotypically and functionally specialized *in vivo* beyond the phenotype of BMDC. The major subsets of DC *in vivo* include CD11b^+^ CD8α^−^, CD11b^−^ CD8α^+^ “lymphoid-resident” DC and B220^+^ plasmacytoid DC. We infected DC purified from the spleens of wild-type mice with VACV expressing EGFP-OVA driven by early or late promoters and examined expression of EGFP-OVA in each of these DC subsets. Expression of eGFP from VACV-eGFP-OVA-Late was not detectable above background levels in infected plasmacytoid DC (CD11c^+^, B220^+^), CD11b^+^ CD8α^−^ DC, or CD11b^−^ CD8α^+^ DC while each DC subset readily expressed eGFP from eGFP-OVA-Early (Fig. 2C). Thus, VACV undergoes an abortive infection in all DC subsets such that VACV late promoter-driven antigens are not expressed following infection.

**Figure 3:**
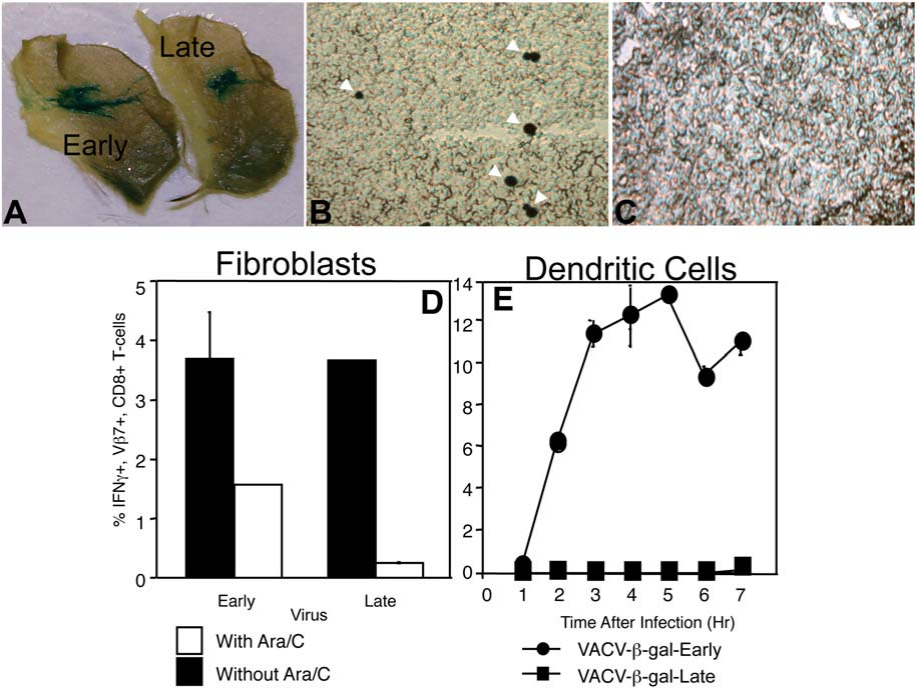
Late promoter-driven β-gal is not produced in pAPC *in vivo* or presented to T_CD8+_ by infected BMDC. Production of β-gal was visualized *in vivo* following i.d. infection in the ear pinnae at the site of infection (A) and draining lymph nodes [(B) Early, (C) Late]. (D) Direct presentation by fibroblasts infected with rVACV-β-gal-Early or rVACV-β-gal-Late was measured by analyzing IFN-γ production from β-gal_96–103_-specific T_CD8+_ in the presence (white bars) or absence (black bars) of ara/c, which will block production of late genes. (E) Similarly, direct presentation by BMDC infected with rVACV-β-gal-Early (•) or rVACV-β-gal-Late (▪) was measured by analyzing IFN-γ production from β-gal_96–103_ specific T_CD8+_.

To extend these observations *in vivo* we infected mice intradermally with rVACV-β-gal-Early or rVACV-β-gal-Late and then visualized β-gal production at the site of infection or in the draining lymph node. Twelve h after infection, β-gal production was readily detectable from either virus at the site of infection (Fig. 3A). However, production of β-gal could only be detected in the draining lymph node after infection with rVACV-β-gal-Early (Fig. 3B,C). We have previously observed that all of the VACV infected cells in a lymph node are macrophages or DC at 12 h post infection [10] indicating that late promoter-driven antigen is undetectable in infected pAPC *in vivo*.

The primary substrates for production of peptides in the direct-presentation pathway are rapidly degraded proteins that may be defective [2]. Such proteins are unlikely to acquire the secondary structure required to become enzymatically active and so may not be detected in our assays. To ensure that β-gal from rVACV-β-gal-Late is not directly presented by virus-infected BMDC, we infected BMDC or fibroblasts expressing H2-K^b^ and measured antigen presentation to primary β-gal_96–103_-specific T_CD8+_. Infected fibroblasts stimulated interferon-γ production in T_CD8+_ regardless of whether the early or late promoter drove β-gal production (Fig. 3D). VACV-infected BMDC triggered interferon-γ by β-gal_96–103_-specific T_CD8+_ only when infected with rVACV-β-gal-Early (Fig. 3E) even when the infection was allowed to proceed for >12 h (data not shown). Thus, direct presentation of β-gal driven by a late promoter did not occur in infected pAPC.

**Figure 4:**
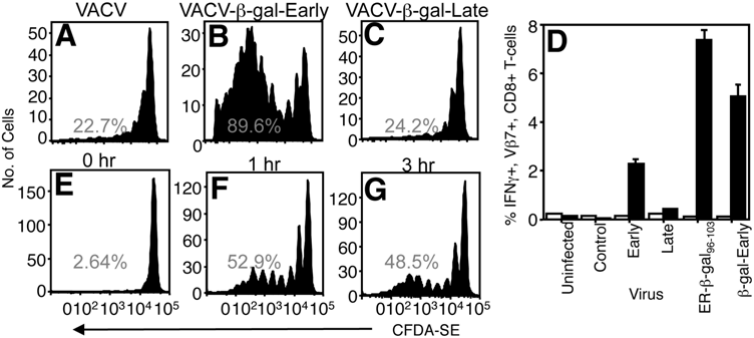
Late VACV promoter-driven antigen is not available for cross presentation. Proliferation (A–C) of adoptively transferred β-gal-specific TCR transgenic T_CD8+_ was measured following immunization with TAg-β_2_m_neg_ cells infected with VACV that does not express β-gal (A), rVACV-β-gal-Early (B), or rVACV-β-gal-Late (C) for 5 h. (D) β-gal_96–103_-specific IFN-γ production by adoptively transferred BG1 T_CD8+_ was measured following immunization with TAg-β_2_m_neg_ cells infected for 5 h with VACV as shown. IFN-γ production is shown in the presence (black bars) or absence (open bars) of β-gal_96–103_ peptide. (E–G) TAg-β_2_m_neg_ cells were infected with rVACV-β-gal-Early for 0 h (E), 1 h (F), or 3 h (G) and assayed for their ability to initiate proliferation of adoptively transferred β-gal-specific TCR transgenic T_CD8+_.

Under conditions where the direct-presentation pathway is blocked *in vivo*, the cross-presentation pathway is thought to compensate and allow generation of T_CD8+_ [11],[12]. However, this compensatory mechanism does not occur with late promoter-driven VACV β-gal (Fig. 1), despite the accumulation of large quantities of antigen that should increase the efficiency of cross presentation [13]. This observation has been interpreted as a functional irrelevance of cross presentation in the induction of virus-specific T_CD8+_ [14], but could also be explained by an inability of late promoter-driven antigen to enter the cross-presentation pathway, a hitherto undescribed phenomenon. To examine cross presentation of β-gal driven by the early or late promoters, we infected SV40 transformed cells that lack β_2_-microglobulin (TAg-β_2_m_neg_) and are therefore direct presentation-incompetent. At 5 h post-infection, a time point at which equivalent levels of β-gal are expressed (Fig. 2A), the cells were treated with psoralen and UVC to halt both protein production and potential virus spread [15]. We measured the ability of these cells to stimulate proliferation and effector function of adoptively transferred BG1 T_CD8+_ following *in vivo* immunization. Under these conditions, initiation of a T_CD8+_ response can only occur following antigen presentation via the cross-presentation pathway. TAg-β_2_m_neg_ cells infected with rVACV-β-gal-Early efficiently triggered proliferation of BG1 T_CD8+_ (Fig. 4B) but those infected with rVACV-β-gal-Late failed to stimulate proliferation (Fig. 4C) or effector function at levels above those found following immunization with TAg-β_2_m_neg_ cells infected with a control VACV (Fig. 4D). Similar data were obtained after infection with rVACV-β-gal-Late for up to 11 h (data not shown), a time point at which p11-driven β-gal is present in enormous excess compared to p7.5 driven β-gal (Fig. 2A). Infection with rVACV-β-gal-Early allowed access to the cross-presentation pathway *in vivo* as soon as 1 h post-infection (Fig. 4E–G) indicating that antigen was not limiting even when present at low intracellular concentrations. These data clearly indicate that late promoter-driven VACV β-gal is not accessible to the cross-presentation pathway even when present in very large quantities.

**Figure 5:**
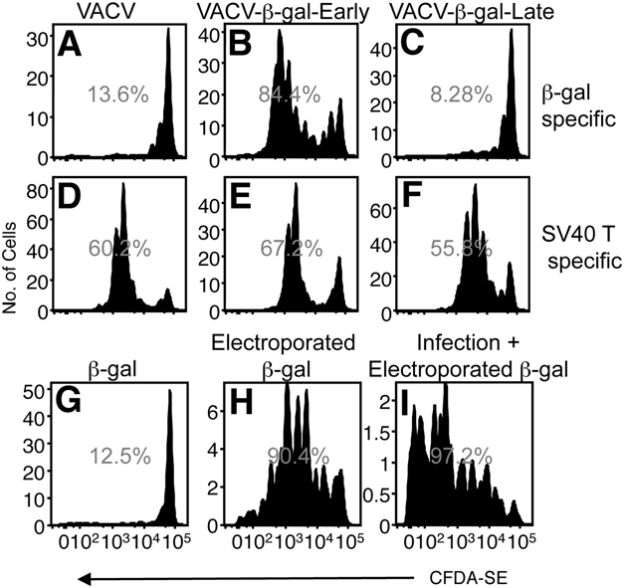
VACV infection does not inhibit the cross presentation of cellular antigen. Proliferation of adoptively transferred CFDA-SE labeled β-gal-specific (A–C) or SV40 TAg Site I-specific (D–F) TCR transgenic T_CD8+_ was measured following immunization with TAg-β_2_m_neg_ cells infected with VACV that does not express β-gal (A,D), rVACV-β-gal-Early (B,E), or rVACV-β-gal-Late (C,F). Proliferation of adoptively transferred β-gal-specific (G–I) TCR transgenic T_CD8+_ was measured following immunization with TAg-β_2_m_neg_ cells incubated with 1 mg/mL β-gal (G), electroporated with 1 mg/mL β-gal (H), or infected with rVACV for 5 h and electroporated with 1 mg/mL β-gal (I).

We have previously demonstrated that cellular protein synthesis, which is rapidly halted following VACV infection, is not required for antigen donation [8]. Nonetheless, it is possible that VACV infection may block donation of all cellular antigen. To investigate this possibility, we exploited the expression of the SV40 T antigen (TAg) as a cellular protein in TAg-β_2_m_neg_ cells. We measured proliferation of adoptively transferred BG1 and SV40 TAg Site I-specific TCR transgenic T cells [16] simultaneously in mice immunized with TAg-β_2_m_neg_ cells infected with rVACV-β-gal-Early or rVACV-β-gal-Late. As before, rVACV-β-gal-Late infected TAg-β_2_m_neg_ cells failed to induce proliferation of BG1 T_CD8+_ (Fig. 5C) but in the same recipient mice proliferation of Site I TAg T_CD8+_ occurred efficiently (Fig. 5F). The entry of cellular antigen into the cross-presentation pathway is therefore not blocked by VACV infection.

It is possible that VACV encoded proteins produced after infection can bind to newly synthesized cellular antigen and prevent entry into the cross-presentation pathway. However, as TAg is constitutively expressed in TAg-β_2_m_neg_ cells the existing cellular pool of antigen could be resistant to such a mechanism of inhibition of cross presentation. Ideally, to examine this possibility one would initiate transcription of a cellular antigen after VACV infection, but as VACV is so adept at shutting down host protein synthesis the initiation of transcription of a cellular gene following VACV infection is technically challenging. Therefore we introduced soluble antigen into TAg-β_2_m_neg_ cells after 5 h of VACV infection and measured the response to this antigen *in vivo*. Again, VACV infection did not inhibit the donation of β-gal (Fig. 5G–I) or OVA (not shown) introduced into infected cells. These data indicate that VACV does not globally suppress the availability of antigen to enter the cross-presentation pathway *in vivo* but utilizes a specialized mechanism to prevent the access of its own antigens to the cross-presentation pathway.

**Figure 6:**
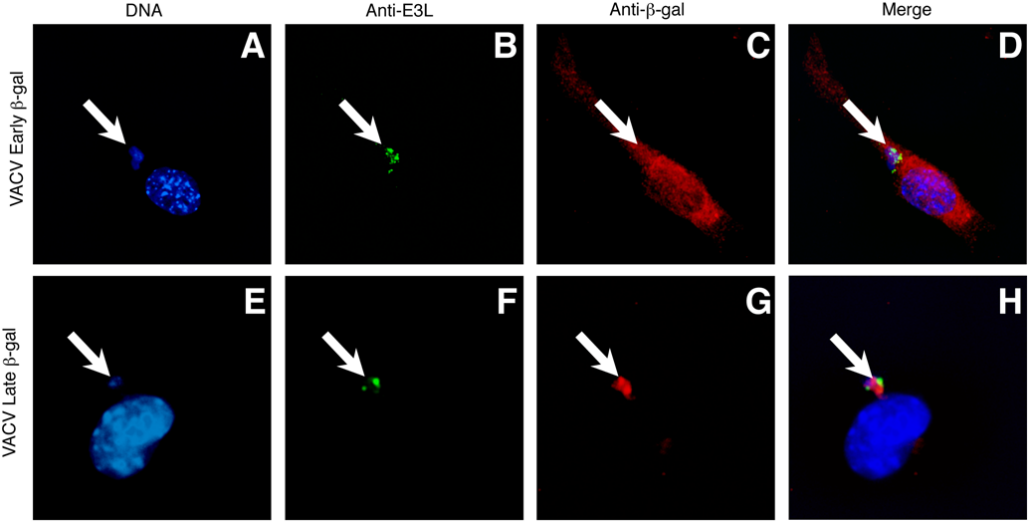
Late antigen that is not available for cross priming is sequestered in VACV viral factories. TAg-β_2_m_neg_ cells were infected with rVACV-β-gal-Early (A–D) or rVACV-β-gal-Late (E–H) for 5 h and stained with antibodies to the VACV protein E3L (B,D,F,H) and β-gal (C,D,G,H). All cells were incubated with the nuclear counterstain DAPI (A,D,E,H). The white arrows indicate the location of viral factories in infected cells.

Katsafanas and Moss recently described that soluble proteins driven by intermediate and late promoters are concentrated within cytosolic virus factories following coordinated transcription and translation within these domains [17]. Virus factories are rough endoplasmic reticulum-bound perinuclear organelles in which VACV replication and early assembly of viral particles occurs [18]. There is a possibility that the specialized structure of these compartments in which late antigens are synthesized could prevent access to the cross-presentation pathway. VACV-infected TAg-β_2_m_neg_ cells were visualized to determine the localization of β-gal relative to virus factories labeled with DAPI and the VACV double stranded RNA binding protein E3L (Fig. 6). β-gal from rVACV-β-gal-Early was distributed throughout the cytosol of the cell (Fig. 6C,D), and only 1.3% (+/−0.2) of pixels staining for β-gal were localized within virus factories. In contrast, β-gal from rVACV-β-gal-Late was localized only to the perinuclear virus factories (Fig. 6G,H), with greater than 83% (+/−4.8%) of pixels staining for β-gal being localized within virus factories. An altered distribution of antigen thereby correlates with an inability of that antigen to enter the cross-presentation pathway, and sequestration of newly synthesized antigen within VACV virus factories likely facilitates this process.

**Figure 7:**
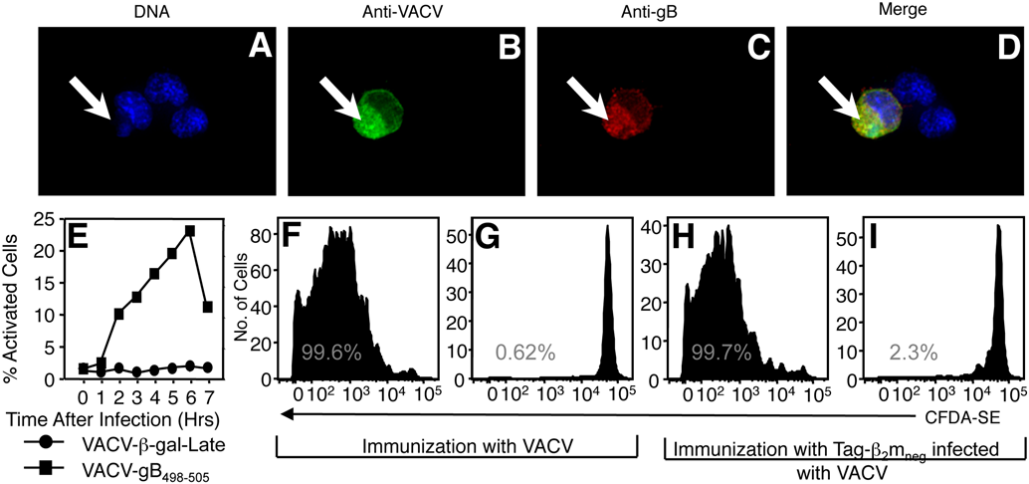
Late VACV promoter-driven antigen that exits virus factories is not directly presented, but is available for cross priming. TAg-β_2_m_neg_ cells were infected with rVACV-gB-Late (A–D) for 5 h, fixed and stained with a polyclonal antisera to VACV (B,D) and a monoclonal antibody to HSV gB (C,D) and the nuclear counterstain DAPI (A,D). The white arrows indicate the location of viral factories in infected cells. (E) Direct presentation to a gB_498–505_ specific T_CD8+_ line was measured following infection of BMDC with rVACV-gB-Late (•) or rVACV-gB_498–505_ (▪). (F–I) Proliferation of adoptively transferred gB_498–505_-specific TCR transgenic T_CD8+_was measured following immunization with rVACV-gB-Late (F), VACV that did not express gB (G), TAg-β_2_m_neg_ cells infected with rVACV-gB-Late (H) or TAg-β_2_m_neg_ cells infected with VACV that did not express gB (I).

To test whether sequestration of antigen within virus factories is essential for the blockade in cross presentation we used recombinant VACV expressing the model antigen HSV-1 glycoprotein B (gB) driven by the p11 promoter (rVACV-gB-Late) [19]. The egress of some late VACV proteins from virus factories is required for viral replication. Targeting of such proteins to the secretory pathway allows proteins to leave the virus factories, so we surmised that similar sequences within the gB protein might allow this protein to exit the factories. Figure 7A–D demonstrates that, in contrast to β-gal driven by a late VACV promoter, gB driven by the identical p11 promoter distributes across many cellular membranes and is not confined to VACV factories. The ability of gB to leave virus factories did not allow direct presentation of the gB_498–505_ peptide by pAPC, as BMDC infected with rVACV-gB-Late did not activate a gB-specific T cell hybridoma (Fig. 7E). However, proliferation of adoptively transferred gB-specific TCR transgenic T_CD8+_ could be detected following immunization with rVACV-gB-Late (Fig. 7F). As direct presentation was blocked in pAPC, the proliferation likely resulted from cross presentation of gB-derived peptides. To test whether gB restricted to the cross-presentation pathway was immunogenic *in vivo* we immunized mice with TAg-β_2_m_neg_ cells infected with VACV-gB-Late for 5 h. In contrast to the results observed with β-gal that was sequestered within VACV factories, TAg-β_2_m_neg_ cells infected with VACV-gB-Late did stimulate proliferation of gB-specific TCR transgenic T_CD8+_ (Fig. 7H). Thus, antigen that can leave VACV factories is available for cross presentation but antigen that remains sequestered within these factories is blocked from entering the pathway.

**Figure 8:**
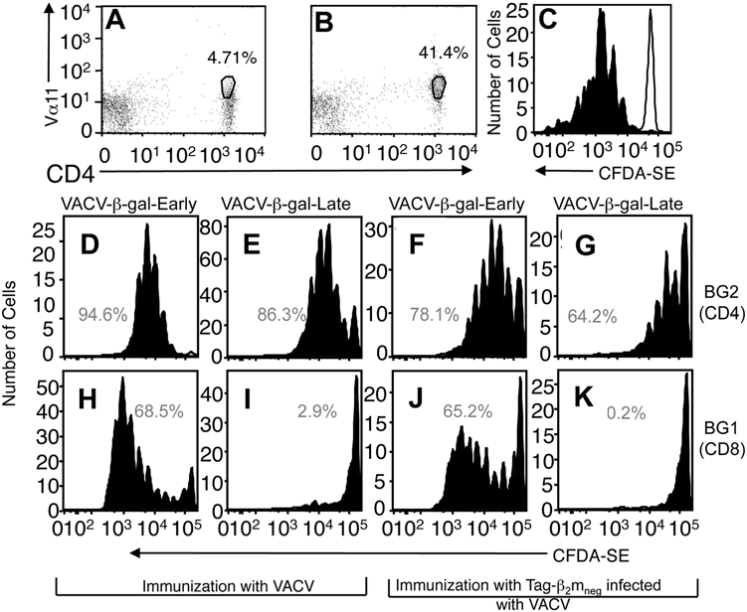
Sequestered antigen is not available for cross priming, but can be presented via the MHC Class II processing pathway. Expression of the Vα11 T cell receptor chain in T_CD4+_ from wild-type (A) or BG2.SJL (B) mice. (C) Division of adoptively transferred BG2.SJL T_CD4+_ following immunization with rVACV-β-gal-Early (black) or a VACV that does not express β-gal (white). Division of adoptively transferred β-gal-specific T_CD4+_ (D–G) or T_CD8+_ (H–K) following immunization with rVACV-β-gal-Early (D,H), rVACV-β-gal-Late (E,I), TAg-β_2_m_neg_ cells infected with rVACV-β-gal-Early (F,J) or TAg-β_2_m_neg_ cells infected with rVACV-β-gal-Late (G,K).

Having gained a mechanistic insight into the means by which VACV acts within the virus infected cell to prevent access of late antigen to the cross-priming pathway we sought to investigate at what point the blockade of cross presentation occurred within pAPC. In order to preserve the *in vivo* nature of our studies we examined presentation of early or late promoter-driven β-gal by the MHC Class II presentation pathway. MHC Class II-mediated presentation of exogenous antigens shares many common components with the MHC Class I-restricted cross-presentation pathway so a differential ability to enter this pathway would give a strong indication of the point at which cross presentation is blocked. In order to study MHC Class II-restricted presentation of β-gal *in vivo* we constructed a transgenic mouse (BG2) bearing a T cell receptor specific for a β-gal peptide presented in complex with MHC Class II. The majority of CD4 cells in the resulting mice expressed the Vα11 chain from the transgene (Fig. 8B) and produced IL-2, IFN-γ and TNF-α in response to peptide sequences corresponding to residues 725–735 from β-gal [20] (Table 1). The T_CD4+_ from the transgenic mice also proliferated following adoptive transfer into a wild-type mouse that was then infected with rVACV-β-gal-Early (Fig. 8C).

**Table 1:** Mapping of the BG2 β-gal-specific T_CD4+_ response.

Peptide #	Antigen	Cytokines (ng/ml)		
		IL-2	IFN-γ	TNF-α
	APC Only	bd	bd	0.007
	No Ag	bd	bd	0.012
	β Gal	0.62+/−0.08	8.61+/−0.76	0.97+/−0.20
123	EAGHISAWQQWRLAEN	bd	bd	0.012
124	SAWQQWRLAENL**SVTLP**	bd	bd	0.013
125	RLAENL**SVTLPAASHAI**	1.16+/−0.22	0.33+/−0.04	0.31+/−0.05
126	**SVTLPAASHAI**PHLTTS	1.33+/−0.31	1.88+/−0.31	0.36+/−0.06
127	**ASHAI**PHLTTSEMDFCI	bd	bd	0.012
128	HLTTSEMDFCIELGNKR	bd	bd	0.011

To map the BG2 determinant, transgenic T cells were incubated with splenocytes in the presence of overlapping peptides (1 µM) or whole βgal (50 µg/ml). Supernatants were collected for cytokine analysis 48 hr post-stimulation using the CBA kit from BD Biosciences. Only the peptides shown stimulated cytokine production by BG2 cells.

MHC Class II-restricted presentation can occur through a number of pathways, including presentation of endogenously synthesized antigen [21]. Early antigen may enter this pathway, but late antigen is not synthesized within pAPC (Fig. 1) and so will not be presented from endogenous sources. To ensure that we were directly comparing MHC Class II-restricted presentation of β-gal driven by early or late promoters we adoptively transferred both BG1.SJL T_CD8+_ and BG2.SJL T_CD4+_ into mice and then immunized with TAg-β_2_m_neg_ cells infected with rVACV-β-gal-Early, rVACV-β-gal-Late or control rVACV as above. We readily detected MHC Class I- and MHC Class II-restricted responses following immunization with rVACV-β-gal-Early or with TAg-β_2_m_neg_ cells infected with rVACV-β-gal-Early (Fig. 8D,F,H,J). As previously shown we did not observe an MHC Class I-restricted response following immunization with rVACV-β-gal-Late (Fig. 8I) or cells infected with rVACV-β-gal-Late (Fig. 8K) but we did detect an MHC Class II-restricted response under both of these circumstances (Fig. 8E,G). Sequestration of antigen, therefore, specifically blocks components of the cross-priming pathway but not the MHC Class II presentation pathway.

## Discussion

The data presented here demonstrate three significant points. First, we show that cross presentation is an important compensatory mechanism of antigen presentation which when blocked results in a complete ablation of the T_CD8+_ response. If a virus inhibits the direct-presentation pathway *in vivo* the resulting T_CD8+_ response is often unchanged [12],[22]. In contrast, we have demonstrated that if entry to the cross-presentation pathway is blocked when the direct-presentation pathway is unavailable, the T_CD8+_ response for the affected antigens is undetectable. Second, although many studies have described the modulation of the direct-presentation pathway, this is the first to describe a viral strategy to evade the cross-presentation pathway. Third, our data demonstrate that the blockade in cross presentation occurs because a number of viral antigens are sequestered within virus factories indicating that the subcellular localization of antigen may prevent access to the cross-presentation pathway. This observation has far reaching implications, as an altered localization of cellular antigens that are normally sequestered from the cross-presentation pathway may allow the induction of T_CD8+_-mediated autoimmunity. The blockade in cross presentation is specific for the cross-presentation pathway, as MHC Class II-restricted presentation of exogenous late antigen is unaffected.

A previous study has examined the impact of altered cellular localization upon donation of antigen during cross presentation. The authors found that cellular localization could affect the efficiency of cross presentation [23] but the study could not rule out an effect of altered antigen stability, a known factor in the effectiveness of cross presentation [3]. Thus, prior investigations have not produced direct evidence to indicate that alteration of the cellular localization of an antigen can enhance or prevent its entry to the cross-presentation pathway. VACV infection alters vesicular trafficking within infected cells and induces the formation of specialized structures such as virus factories. VACV virus factories are cytoplasmic structures that are bound by rough ER. The ER membrane surrounding VACV virus factories is not continuous, however, and “holes” to the cytosol do exist [18]. Intermediate and late VACV proteins are transcribed and translated within virus factories [17] and require specialized signals to leave these structures [24]. The rules governing exit from VACV virus factories remain to be fully characterized. In our current study identical antigens with different cellular localizations are presented differently, with cross presentation of those sequestered within viral factories being completely ablated whereas those that are localized to the cytosol are available for cross presentation. This could indicate that alteration of the localization of cellular antigen may also prevent the entry of antigen into the cross-presentation pathway and subsets of the cellular proteome could be unavailable to the cross-presentation pathway. Point mutations in motifs responsible for the targeting of protein to compartments that sequester antigen from the cross-presentation pathway would render these antigens immunogenic, potentially producing T_CD8+_-mediated autoimmunity via the cross-presentation pathway.

The blockade in cross presentation is specific, as the MHC Class II pathway that shares many components with the cross-presentation pathway is unaffected. Thus, pAPC-mediated internalization and degradation of late antigens sequestered within virus factories is likely unaltered. As MHC Class I-restricted direct presentation of late antigens sequestered within virus factories readily occurs this strongly indicates that the mechanism involved targets a specific component of the cross-presentation pathway. The unique component of the cross-presentation pathway involves release of antigen from within an endosomal/lysosomal compartment into the cytosol [25],[26], a process that may involve the retrotranslocation machinery involved in ER-associated degradation [27]. Human Cytomegalovirus alters ER-associated degradation to increase the degradation of MHC Class I heavy chains within infected cells, so the manipulation of this degradative pathway by viruses is possible [28]. Cross presentation of β-gal derived from VACV-β-gal-Early requires the TAP transporter (data not shown), and thus retrotranslocation into the cytosol. This process of release of antigen into the cytosol represents the likely mechanism responsible for blockade of the cross-presentation pathway.

Our studies have utilized model antigens expressed by VACV but the observations made can readily be extended to native VACV antigens. A number of studies have mapped MHC class I-restricted antigenic determinants from VACV proteins restricted by either mouse [29],[30],[31] or human MHC molecules [32],[33],[34]. The source of the mapped determinants reveals that the majority of peptides recognized are derived from early VACV gene products. In contrast, the majority of MHC Class II-restricted determinants are found within late VACV gene products [35]. A small number of peptides recognized by T_CD8+_ are found in late genes. All of these immunogenic late VACV genes contain N-terminal signal sequences or hydrophobic transmembrane domains and are components of the intracellular mature virus, intracellular enveloped virus, or extracellular enveloped virus membranes that would leave virus factories. The remainder of the determinants mapped within late VACV gene products are present within proteins that may associate with other VACV proteins (e.g. A10L that associates with A4L [36]) to facilitate their exit from factories. These data validate our hypothesis that late VACV proteins that remain within virus factories are not immunogenic whereas those that can leave can generate T_CD8+_ responses, likely via the cross-presentation pathway.

Peptides derived from late gene products can enter the direct-presentation pathway, irrespective of whether the protein from which they are derived cannot exit the virus factory (Fig. 3D). However, late VACV gene products are not produced within infected pAPC, and so any immunogenicity in the T_CD8+_ compartment likely results via the cross-presentation pathway. VACV is closely related to the cowpox virus [37], which has been demonstrated to inhibit direct presentation by inhibiting movement of peptide-loaded MHC Class I molecules out of the ER [38],[39]. It is not beyond the realm of possibility that a common ancestor of cowpox virus and VACV inhibited MHC Class I-restricted presentation of the majority of virus proteins. If egress of a particular late protein was required for virus replication then presentation of that antigen via the cross-presentation pathway could be evolutionarily tolerated. However, VACV has clearly gone to significant lengths to prevent access of other antigens to the cross-presentation pathway producing a newly discovered mechanism of evasion of the adaptive immune response.

## Materials and Methods

### Animals

Female C57BL/6 mice were purchased from Charles River Laboratories (Wilmington, MA). OT-1 TCR RAG1^−/−^ transgenic mice [40],[41] were obtained from the NIAID Exchange Program (Line 4175). gBT-1.3 mice were a kind gift from Dr. Frank Carbone (University of Melbourne, Victoria, Australia) [42]. B6.SJL-Ptprca/BoAiTac mice were purchased from Taconic Farms (Germantown, NY) and bred to both OT-1 TCR and BG1 TCR mice to produce OT-1.SJL and BG1.SJL offspring, respectively. SV40 Site I TCR mice were a kind gift from Dr. Satvir Tevethia (Milton S. Hershey Medical Center, Hershey, PA) [16]. All mice were maintained under specific pathogen-free conditions at the M. S. Hershey Medical Center. All studies were approved by the Penn State College of Medicine Institutional Animal Care and Use Committee.

### Development of BG2 TCR Transgenic Mice

BG2 mice that express a T cell receptor on T_CD4+_ specific for an MHC class II-I-A^b^-restricted epitope of β-gal on a C57BL/6 background were generated. Total RNA was isolated from an I-A^b^-restricted, β-gal specific T_CD4+_ clone and the α and β TCR were amplified by a 5′-Rapid Amplification of cDNA Ends (5′ RACE, Invitrogen, Carlsbad, CA) using constant region anti-sense primers a1 (5′-GGCTACTTTCAGCAGGAGGA-3′) and b1 (5′-AGGCCTCTGCACTCATGTTC-3′), respectively. 5′-RACE products were amplified with nested TCR alpha and beta constant region primers a2 (5′-GGGACTCAAAGTCGGTGAAC-3′) and b2 (5′-CCACGTGGTCAGGGAAGAAG-3′) and cloned into pCR4TOPO TA sequencing vectors (Invitrogen). Genomic cloning PCR primers were designed based upon the method previously described [43]. The genomic variable domains were validated by sequencing, subcloned into TCR cassette vectors kindly provided by Dr. Diane Mathis (Harvard), and coinjected into fertilized C57BL/6 embryos (SAIC, Frederick, MD) yielding TCR transgenic founder mice. Mice were bred with B6.SJL mice and maintained as heterozygotes. Transgene expression monitored by PCR or by staining of blood cells. For PCR, tail samples from 3–4 week old mice were employed for genotyping of BG2 mice using the red Extract-N-Amp Tissue PCR kit (Sigma, St. Louis, MO). Primers used are as follows: BG2 Alpha F1: ACAACCCGGGATTCCACAG; BG2 Alpha R1: GTATAGCGGCCGCCTCCTAGTGCAATGGT; BG2 Beta F1: TATCTCGAGTCCTGCCGTGACCCTACTATG; BG2 Beta R1: CAGCCGCGGAACCCAACACAAAAACTATAC.

Transgene expression was monitored by flow cytometry following staining with anti-PE-Vα11 (Clone RR8-1) and anti-PE-Cy5-CD4 (Clone L3T4) antibodies. To map the BG2 determinant, transgenic T cells were incubated with splenocytes in the presence of overlapping peptides (1 µM) or whole βgal (50 µg/ml). Supernatants were collected for cytokine analysis 48 h post-stimulation using the CBA kit from BD Biosciences (San Jose, CA). Only the peptides shown in Table 1 stimulated cytokine production by the BG2 cells.

### Viruses

VACV (Western Reserve strain), rVACV-β-gal-Late, rVACV-β-gal-Early, rVACV-gB-Late, rVACV-OVA, rVACV-gB_498–505_, rVACV-CD4 [44] and recombinant adenovirus expressing β-gal (Ad-β-gal) were a kind gift from Dr. Jon Yewdell and Dr. Jack Bennink (Laboratory of Viral Diseases, NIAID, Bethesda, MD). VACV expressing the β-gal_96–103_ peptide (rVACV-β-gal_96–103_) targeted to the endoplasmic reticulum (ER) with a signal sequence derived from the adenovirus E3/19k protein was previous published [45].

### Generation of VACV-eGFP-OVA Constructs

The plasmid pRB21 expressing the full length vp37 VACV ORF with the p7.5 early/late promoter was a kind gift from Dr. Bernard Moss (Laboratory of Viral Diseases, NIAID, Bethesda, MD) [46]. The peGFP-C1 plasmid expressing full-length OVA (peGFP-C1-OVA_1–385_) was a kind gift from Dr. Kenneth Rock (Department of Pathology, University of Massachusetts Medical School, Worcester, MA) [23]. For construction of VACV-eGFP-OVA-Late pRB21 backbone DNA was ligated with eGFP-OVA using T4 DNA Ligase (Invitrogen). Following ligation, plasmid DNA was sequenced to ensure that the vp37, p7.5 early/late promoter, and eGFP-OVA_1–385_ sequences were correct. To make rVACV-eGFP-OVA-Late the p11 promoter was inserted in place of the p7.5 promoter. rVACV-eGFP-OVA-Early and rVACV-eGFP-OVA-Late were generated by infecting transfected BSC-1 cells infected with VACV-vRB12 at an MOI of 1 using the CellPhect Transfection Kit (GE Healthcare, Buckinghamshire, UK). As VACV-vRB12 contains the flanking sequences of vp37, homologous recombination occurred to allow virus spread [46]. The resulting rVACV were plaque purified three times prior to characterization. The resulting rVACV-eGFP-OVA-Early and rVACV-eGFP-OVA-Late produced green fluorescence upon infection of WT3 cells and sequencing revealed the presence of the correct promoter and OVA sequences in DNA purified from virions.

### Cell Lines and Cultures

All media were purchased from Invitrogen. WT3 [47], TAg-β_2_m_neg_ [15] and L929 fibroblasts that stably express K^b^ (L-K^b^) were maintained in Dulbecco's Modified Eagle Media containing 10% fetal bovine serum (FBS) supplemented with penicillin/streptomycin and 2 mM L-glutamine. E22 cells (the H2^b^ EL4 thymoma transfected with β-gal) [45] were maintained in RPMI 1640, 5% FBS, penicillin/streptomycin, 2 mM L-glutamine and 400 mg/ml G418. The gB_498–505_-specific LacZ T cell hybridoma, 2E2, was a kind gift from Dr. Frank Carbone (University of Melbourne, Victoria, Australia) and was maintained in RPMI 1640, 5% FBS, penicillin/streptomycin, 2 mM L-glutamine.

Bone marrow-derived dendritic cells (BMDC) were generated as previously described [48].

### DC Isolation

C57BL/6 mice were inoculated i.d. with approximately 5×10^5^ Flt3 ligand expressing B16 tumor cells. Two weeks later the spleens from immunized mice were harvested, microdissected, and incubated in 1 mg/mL Collagenase D (Roche Diagnostics, Indianapolis, IN) at 37°C for 20 min. Following lysis of red blood cells the remaining cells were incubated with Pan-DC microbeads (Miltenyi Biotec, Auburn, CA) and positively sorted. Purified DC were infected with rVACV-eGFP-OVA_1–385_-Early or rVACV-eGFP-OVA_1–385_-Late at an MOI of 10 for a duration of 7 hours in the presence or absence of cytosine arabinoside and analyzed by flow cytometry for the expression of eGFP.

### T Cell Culture

Live mononuclear splenocytes from mice immunized 30 d previously with 1×10^6^ pfu Ad-β-gal were harvested by centrifugation over a Lymphocyte Separation Medium (LSM) cushion (BioWhittaker, Walkersville, MD), washed once and resuspended at 1×10^7^ cells per well in RPMI 1640 with 10% FBS, 1% non-essential amino acids, penicillin/streptomycin, 2 mM L-glutamine, and 7.5 U/ml of IL-2 (Peprotech, Rocky Hill, NJ). Cells were stimulated weekly with 2.5×10^5^ irradiated E22 cells per well.

### Adoptive Transfer of TCR Transgenic Cells

Spleens and lymph nodes were removed, homogenized to produce a single cell suspension, and mononuclear cells isolated as above. Where indicated, cells were labeled with 5 µM 5-(and-6) carboxyfluorescein diacetate, succinimidyl ester (CFDA-SE, Invitrogen) for 10 min at 37°C and washed once prior to injection.

### Electroporation

Approximately 4×10^6^ TAg-β_2_m_neg_ cells were suspended in phosphate buffered saline (PBS) containing 1 mg/mL ovalbumin (OVA) or 1 mg/mL β-gal with 10 mM MgCl_2_ and incubated on ice for 10 minutes. The cells were then electroporated in disposable cuvettes (Bio-Rad, Hercules, CA) on a Bio-Rad gene pulser at 0.25 kV or 0.45 kV with a capacitance of 250 uFD. Following electroporation, cells were incubated on ice for an additional 10 min and washed three times with 10% Iscoves Modified Dulbecco's Medium (IMDM). Cells were irradiated at 20,000 rad prior to injection.

### *In Vivo* Cross Presentation

For *in vivo* immunization, mice were infected i.v. with 1×10^7^ pfu of VACV or were injected i.p. with TAg-β_2_m_neg_ that were either infected with VACV or electroporated with antigen as described above. TAg-β_2_m_neg_ were infected with VACV at a multiplicity of infection of 10 and then treated with psoralen and ultraviolet light (UV-C) as previously described [3]. As VACV will not infect all cells, in some experiments TAg-β_2_m_neg_ were infected with rVACV-CD4, and infected cells were sorted using anti-CD4 microbeads (Miltenyi Biotech).

### Intracellular Cytokine Staining

Mononuclear cells isolated from splenocytes or T_CD8+_ lines were washed twice after isolation over an LSM cushion and plated in triplicate into individual wells of a 96 well plate (3×10^6^ cells per well). Cells were stimulated with 10^−6^ β-gal_96–103_ peptide for 2 h at 37°C or were incubated with BMDC infected with VACV as indicated. After 2 h of stimulation, 10 µg/mL Brefeldin A (BFA, Sigma, St. Louis, MO) was added and the cells were incubated for another 4 h. T_CD8+_ were then assayed for production of IFN-γ by flow cytometry.

### *In Vitro* Antigen Presentation

BMDC were incubated with anti-CD11c microbeads (Miltenyi Biotech) and positively sorted. Purified DC were infected with VACV (MOI = 20) for a duration of 7 h in the presence or absence of cytosine arabinoside. Infected BMDC were then incubated with β-gal_96–103_-specific T cells generated as outlined above, and activation of the T cells was determined either by intracellular cytokine staining, or by activation of the LacZ hybridoma 2E2 using the chlorophenol red β-D-galactopyranoside (CPRG) substrate of β-gal as outlined below.

### Flow Cytometry

For all assays, cells were incubated on ice with Fc block containing 20% normal mouse serum (Sigma) for 20 min prior to staining. For intracellular cytokine staining analysis, all antibodies were purchased from BD Biosciences except where noted. Cells were stained with anti-CD8 PE-Cy5 (Clone 53-6.7), washed once with PBS, and fixed with 1% paraformaldehyde (PFA). Fixed cells were then stained with anti-IFN-γ-FITC (Clone XMG1.2) in 0.5% saponin, washed, and analyzed. Antibodies used to identify OT-1.SJL or BG1.SJL cells were anti-CD45.1-PE (Clone A20). Antibodies used to identify gBT-I.3 cells were anti-Vα2-PE (Clone B20.1). For SV40 Site I and BG1.SJL double adoptive transfers, cells were stained in triplicate with anti-CD8-PE-Cy7 (Clone 53-6.7) and anti-Vβ7-PE (Clone TR310) for SV40 site I TCR cells and anti-CD45.1-PE-Cy5 (eBioscience, San Diego, CA, Clone A20) for BG1.SJL TCR cells. For BG2 and BG1 double adoptive transfer cells were stained with anti-CD45.1-PE to identify adoptively transferred cells and with anti-CD8-Alexa Fluor 750 and anti-PE-Cy5-CD4 (Clone L3T4) to distinguish the two cell populations. Antibodies used to distinguish DC subsets were anti-CD11c-PE (eBioscience, Clone N418), anti-CD8α-PerCP-Cy5.5 (Clone 53-6.7), anti-CD11b-Alexa Fluor 750 (eBioscience, Clone M1/70), anti-CD45R/B220-Alexa Fluor 647 (eBioscience, Clone RA3682), anti-CD90.2-Biotin (eBioscience, Clone 53-2.1), anti-NK1.1-Biotin (eBioscience, Clone PK 136), anti-CD19-Biotin (eBioscience, Clone 1D3), and PE-Cy7 Conjugated Streptavidin. DC subsets were distinguished based on the expression of CD11c (CD11c^+^, CD8^+^, CD11b^−^, B220^−^) (CD11c^+^, CD8^−^, CD11b^+^, B220^−^) (CD11c^+^, B220^+^) and the lack of expression of CD90.2, NK1.1, and CD19.

### Assays for β-Gal Activity

To measure expression of β-gal, cells were infected with VACV for 1–12 h at a MOI of 10 in IMDM. Activity of β-gal in cells was determined using either of the β-gal substrates, o-nitrophenol β-D-galactoside (ONPG) or CPRG. Briefly, for the ONPG assay, approximately 3–5×10^5^ cells were lysed with 150 µL 1% Igepal (Sigma, St. Louis, MO) and 10 µl aliquots incubated with 150 µL 1 mg/mL ONPG substrate in Z buffer (0.06 M Na_2_HPO_4_, 0.04 M NaH_2_PO_4_, 0.01 M KCl, 0.001 M MgSO_4_, 40 mM β-mercaptoethanol) for 10 min at 37°C. After 10 minutes the reaction was stopped by addition of 50 µL Na_2_CO_3_. β-gal activity was measured using a micro-plate reader (Dynex, Chantilly, VA) at 405 nm wavelength. For the CPRG assay 1×10^5^ cells per well were washed twice in cold PBS and incubated with 0.15 mM CPRG, 10 mM phosphate buffer, 1 mM MgCl_2_, and 0.1255% Igepal. Upon color change, 50 µL of stop buffer (300 mM glycine, 15 mM EDTA, 10 M NaOH) was added, and absorbance measured at a wavelength of 595 nm, with 630 nm as a reference wavelength.

### Intracellular Fluorescence

To measure localization of virally expressed recombinant antigen, TAg-β_2_m_neg_ cells were plated in 8 well Permanox chamber slides (Nalge Nunc International, Rochester, NY) and allowed to adhere overnight. Cells were infected at a MOI of 20 with VACV for 5 h and then fixed for 15 min with 4% PFA. Cells were permeabilized with 0.2% Triton X-100 (Bio-Rad) and blocked with 20% goat serum (Sigma) for 20 min. Infected cells were stained with primary antibodies as follows in 10% goat serum: Unconjugated polyclonal rabbit anti-β-gal IgG antibody (AbCam, Cambridge, MA), mouse anti-vaccinia E3L (TW2.3 supernatant) [49], unconjugated mouse anti-gB IgG antibody (Virusys, Sykesville, MD) or polyclonal rabbit anti-vaccinia IgG-FITC antibody (Biogenesis, Kingston, NH). Secondary antibodies used were goat anti-rabbit IgG-Alexa Fluor 647, goat anti-mouse IgG-Alexa Fluor 647, and goat anti-mouse IgG-Alexa Fluor 488 (all from Invitrogen). The slides were overlaid with ProLong Gold antifade reagent with 4′-6-diamidino-2-phenylindole (DAPI) (Invitrogen) and allowed to cure overnight.

### Ear and Lymph Node Sections

Mice were infected i.d. in each ear with rVACV-β-gal-Early or rVACV-β-gal-Late. Twelve h post-infection, ears were removed and fixed in 2% PFA/0.2% gluteraldehyde. Cervical lymph nodes were frozen in Tissue-Tek OCT Compound (Fisher Scientific, Pittsburgh, PA), sections (15 µm) cut using a Bright Cryostat (Hacker Instruments, Winnsboro, SC) and then fixed with 10% buffered formalin phosphate. β-gal expression was visualized using 5-bromo-4-chloro-3-indolyl-β-D galactopyranoside (X-gal, 0.25 mg/ml) in 2 mµ potassium ferrocyanide, 5 mM ferricyanide and 2 mM MgCl_2_ in PBS following overnight incubation at 37°C.

### Microscopy

All images of infected cells, murine ear and lymph node sections were acquired on an Olympus IX81 deconvolution microscope (Olympus, Center Valley, PA) using Slidebook 4.0 software (Intelligent Imaging Innovations, Denver, CO) or Q Capture software (QImaging, Burnaby, BC, Canada). Colocalization was measured using the Colocalization Plugin for ImageJ analysis software (NIH).

## Supporting Information

Figure S1 β-gal activity limit of detection using a CPRG assay. β-gal protein was titrated from 10^−4^ mg/mL to 10^−12^ mg/mL, and a CRPG assay was used to determine the limit of detection of β-gal activity. Our limit of detection of β-gal activity was 10^−8^ mg/mL of β-gal protein with no activity detected at 10^−9^ mg/mL of β-gal protein.
(0.73 MB TIF)
