# Supplementary figures and images for: Viral Sequestration of Antigen Subverts Cross Presentation to CD8+ T Cells
Source: PLoS Pathog. 2009 May 29;5(5):e1000457. doi: 10.1371/journal.ppat.1000457 (PMC2680035; doi:10.1371/journal.ppat.1000457)

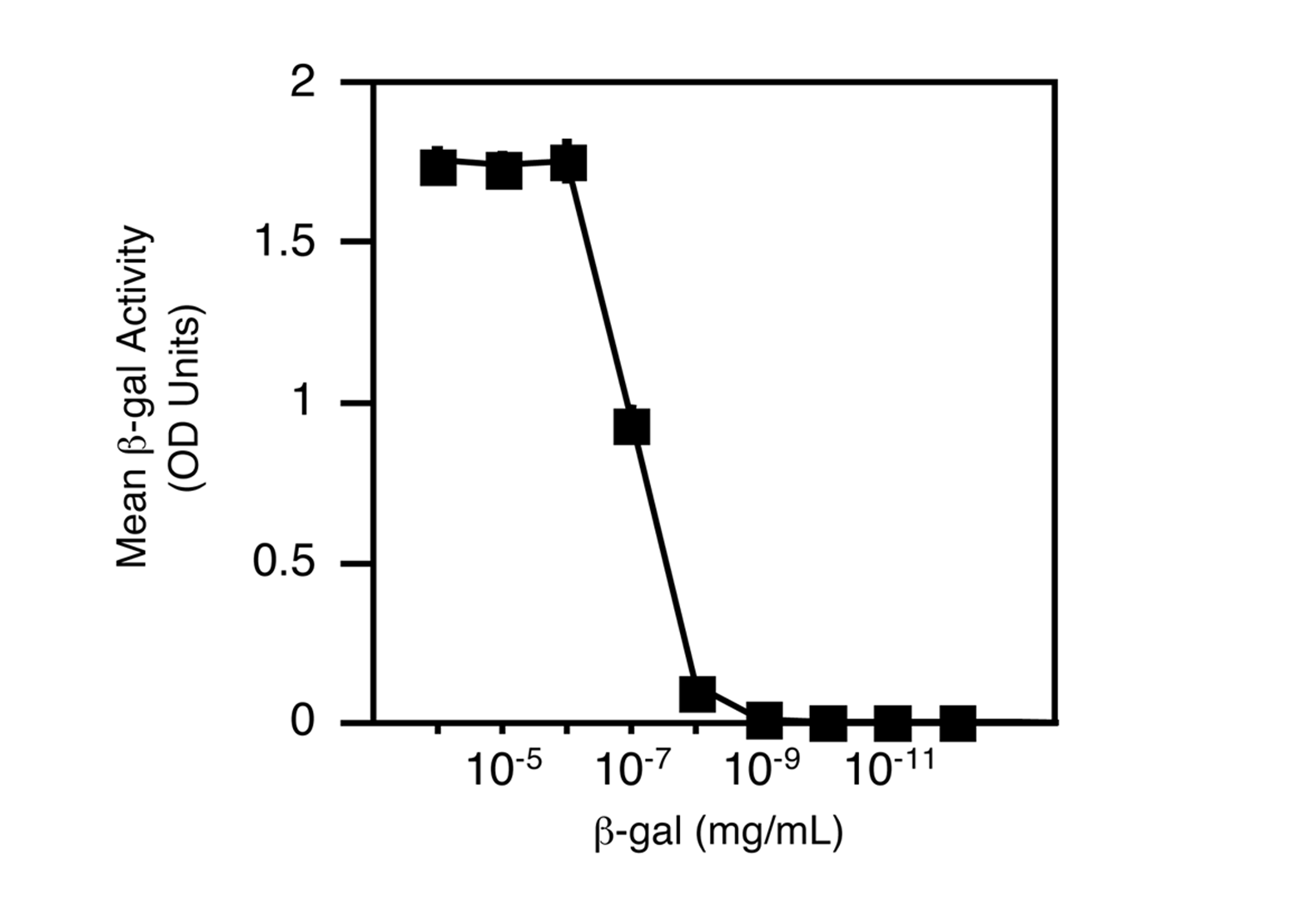

Supplement: Figure S1 — β-gal activity limit of detection using a CPRG assay. β-gal protein was titrated from 10−4 mg/mL to 10−12 mg/mL, and a CRPG assay was used to determine the limit of detection of β-gal activity. Our limit of detection of β-gal activity was 10−8 mg/mL of β-gal protein with no activity detected at 10−9 mg/mL of β-gal protein. (0.73 MB TIF) [file ppat.1000457.s001.tif]
